# Target Cell Cyclophilins Facilitate Human Papillomavirus Type 16 Infection

**DOI:** 10.1371/journal.ppat.1000524

**Published:** 2009-07-24

**Authors:** Malgorzata Bienkowska-Haba, Hetalkumar D. Patel, Martin Sapp

**Affiliations:** Department of Microbiology and Immunology and Feist-Weiller Cancer Center, Louisiana State University Health Sciences Center, Shreveport, Louisiana, United States of America; University of Wisconsin-Madison, United States of America

## Abstract

Following attachment to primary receptor heparan sulfate proteoglycans (HSPG), human papillomavirus type 16 (HPV16) particles undergo conformational changes affecting the major and minor capsid proteins, L1 and L2, respectively. This results in exposure of the L2 N-terminus, transfer to uptake receptors, and infectious internalization. Here, we report that target cell cyclophilins, peptidyl-prolyl cis/trans isomerases, are required for efficient HPV16 infection. Cell surface cyclophilin B (CyPB) facilitates conformational changes in capsid proteins, resulting in exposure of the L2 N-terminus. Inhibition of CyPB blocked HPV16 infection by inducing noninfectious internalization. Mutation of a putative CyP binding site present in HPV16 L2 yielded exposed L2 N-terminus in the absence of active CyP and bypassed the need for cell surface CyPB. However, this mutant was still sensitive to CyP inhibition and required CyP for completion of infection, probably after internalization. Taken together, these data suggest that CyP is required during two distinct steps of HPV16 infection. Identification of cell surface CyPB will facilitate the study of the complex events preceding internalization and adds a putative drug target for prevention of HPV–induced diseases.

## Author Summary

Human papillomaviruses (HPV), especially HPV types 16 and 18, are a major cause of cancer in women worldwide. HPV16, like most genital HPV types, relies on heparan sulfate proteoglycans (HSPGs) to attach to host cells and to the extracellular matrix. Attachment is mediated by surface-exposed basic residues of the major capsid protein, L1. This triggers conformational changes affecting L1 and the minor capsid protein, L2. However, it is not known what interaction triggers these structural changes and if any host cell protein is involved. Now we have identified a host cell chaperone, Cyclophilin B (CyPB), as essential for efficient HPV16 and HPV18 infection. CyPB, which is present on the cell surface in association with specific forms of *O*-sulfated HSPG as well as in the lumen of intracellular membrane structures, is an energy-independent enzyme, which catalyzes cis/trans isomerization of peptidyl-prolyl bonds. We demonstrate that CyPB facilitates conformational changes resulting in exposure of the L2 N-terminus, which is required for infectious entry. In addition, we present some evidence suggesting that members of the cyclophilin family are required for a second, probably intracellular, step of HPV16 infection. This is the first report implicating cell surface chaperones as essential host factors for viral infection.

## Introduction

Cyclophilins (CyP) comprise a family of peptidyl-prolyl cis/trans isomerases, which are evolutionarily conserved and ubiquitously expressed [1],[2]. CyP facilitate folding of nascent proteins and through this have been implicated in RNA splicing, stress responses, gene expression, cell signaling, mitochondrial function, and regulation of kinase activity [3]. The 16 human family members differ mainly by terminal extensions, which are probably responsible for subcellular localization and protein-protein interactions, and by tissue specific expression. CyP were initially identified as high affinity binding proteins for cyclosporin A (CsA), an immunosuppressive agent [4]. CsA blocks the enzymatic acitivity of CyP. Cyclophilin A and B (CyPA and CyPB) are the most abundant among the family, where CyPA mainly localizes to the cytoplasm and CyPB, which encodes a signal peptide, is associated with the endoplasmic reticulum (ER). CyPB can be secreted and is detected on the cell surface, where it colocalizes with heparan sulfate proteoglycans (HSPGs) like syndecan-1 [5]. Recent reports suggest that CyPB preferentially bind HSPG molecules that carry a 3-*O*-sulfated *N*-unsubstituted glucosaminoglycan residue in the heparan chain [6]. 3-*O*-sulfation is the least abundant modification of heparan sulfate and thus only few HSPG molecules on the cell surface are associated with CyPB. The core protein required for triggering biological function of cell surface CyPB is most likely syndecan-1 [5].

Several viruses exploit CyP for life cycle completion. The capsid protein of human immunodeficiency virus type 1 (HIV-1) harbors a CyPA binding site resulting in the incorporation of this chaperone into the virion [7]. In addition, target cell CyPA is required for efficient infection of human cells [8],[9]. Inhibition of CyPA prevents the transport of reverse transcribed viral genome to the nucleus without interfering with reverse transcription [10]. A number of observations were interpreted as CyPA preventing the interaction of the viral capsid protein with restriction factors rather than it promoting viral uncoating. In some nonpermissive cells, CyPA activity is required for binding of the restriction factor TRIM5 to the capsid protein (for review see [7]). Hepatitis C virus (HCV) is another example requiring CyPB activity for efficient replication. It interacts with the viral polymerase NS5B thus promoting RNA binding [11]. Furthermore, mouse cytomegalovirus (MCMV) infection of neural stem/progenitor cells is facilitated by CyPA by an unknown mechanism [12].

Here we demonstrate that CyPB activity facilitates infection of human papillomavirus type 16 (HPV16) and HPV18. HPV are non-enveloped epitheliotropic DNA viruses with a circular, chromatinized, double stranded DNA genome of approximately 8000 bp. They induce benign lesions of the skin and mucosa that in some instances progress to malignancies. HPV induced malignancies, including cervical carcinoma, contribute to more than 7% of all cancers in women worldwide. The viral capsid is composed of 360 copies of the major capsid protein, L1, and up to 72 copies of the minor capsid protein, L2 [13]–[15]. L1 protein, which is organized in 72 pentamers, called capsomeres, mediates the primary attachment of viral particles to the cell surface [16]–[18] and/or extracellular matrix (ECM) of susceptible cells [19], most probably via HSPG [20]. The need for HS can be bypassed by treatment of immature HPV16 pseudovirions with furin convertase [21]. The primary attachment is mediated by surface-exposed lysine residues located at the rim of capsomeres [22]. HPV33 binding to the cell surface requires *O*-sulfation of HS, whereas both *N*- and *O*-sulfation are needed for HSPG to function as an initiator of the infectious entry pathway [23]. These data suggested that secondary HSPG interactions may play a role in infection, which was recently supported by the use of the HS binding drug DSTP-27 [18]. Virus attachment triggers conformational changes in both capsid proteins [23]–[25], which seem to be required for transfer to putative secondary receptors and infectious internalization [18]. Conformational changes result in the exposure of the N-terminus of L2 protein, which contains a highly cross-reactive neutralizing epitope, and subsequent cleavage of 12 N-terminal amino acids catalyzed by furin convertase [24],[26]. Data presented below suggest that cell surface CyPB facilitates exposure of the L2 N-terminus, which is required for infectious internalization.

## Results

### Effect of CyP inhibition on HPV16 and HPV18 infection

**Figure 1:**
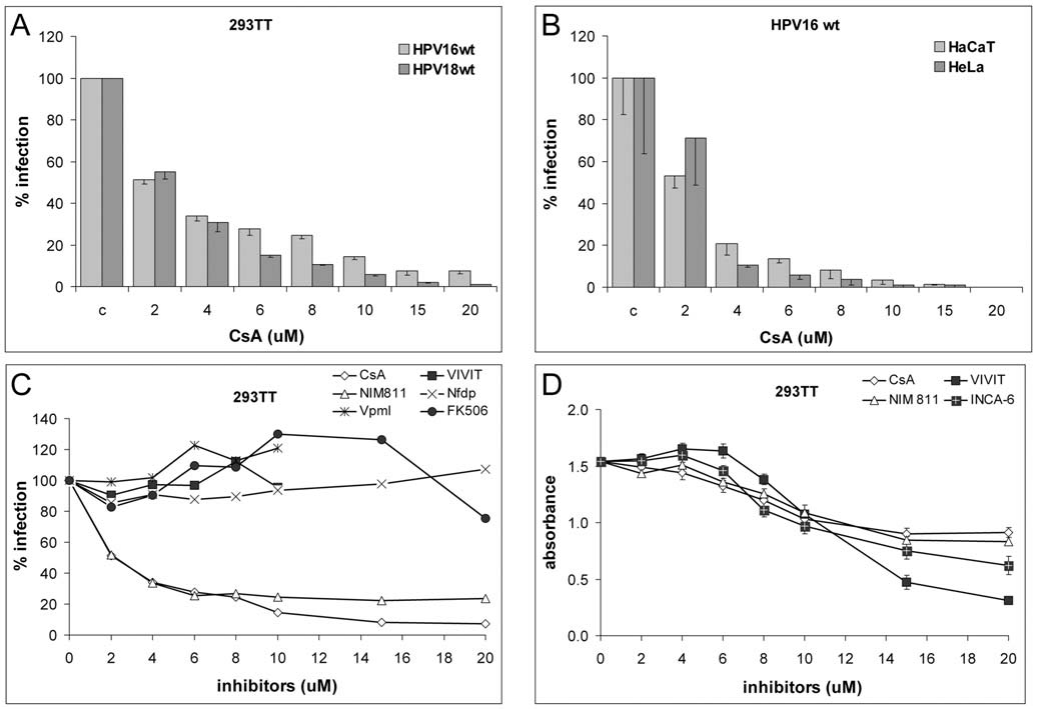
CyP facilitate HPV infection. 293TT (A), HaCaT (B), or HeLa cells (B) were infected with HPV16 (A,B,C) or HPV18 pseudovirus (B) in presence of indicated inhibitors and infection was scored at 72 hpi. (D) Effect of drugs on cell growth was determined by the MTT assay. We did not notice significant increase in cell death. CsA: cyclosporine A; VIVIT: cell permeable 11R-VIVIT; Nfdp: nifedipin; Vpml: verapamil. Representative graphs are based on three replicates each with standard deviation indicated.

We used a well established pseudovirus system for our studies, which relies on the expression of codon-modified forms of L1 and L2 in human embryonic kidney 293TT cells harboring a high copy number packaging plasmid [27]. We packaged a green fluorescent protein (GFP)–based marker plasmid that has been successfully used before to study early events of HPV infection [28]–[31]. We observed that CsA efficiently blocked HPV16 infection of 293TT cells with an inhibitory concentration 50 (IC_50_) of approximately 2 µM (Figure 1A). Similar results were obtained for HaCaT, which is currently the most commonly used keratinocytes-derived cell line for analysis of HPV infection, and HPV-harboring HeLa (Figure 1B). CsA has been shown to block activity of calcineurin and CyP as well as P-glycoproteins, also known as ABC transporters. We used more specific inhibitors to narrow down the cellular target responsible for the observed inhibition. Neither INCA-6 nor the cell permeable R-VIVIT peptide and FK506, inhibitors of the interaction between calcineurin and nuclear factor of activated T cells (NFAT), blocked infection (Figure 1C). Similarly, Virapamil and nifedipine, specific inhibitors of P-glycoproteins, had no effect. In contrast, NIM811, which blocks both P-glycoproteins and CyP, inhibited HPV16 infection as efficiently as CsA. Identical results were obtained for key inhibitor NIM811 in HaCaT cells (data not shown). All inhibitors reduced cell growth of 293TT (Figure 1D), HaCaT and HeLa (not shown) cells to a similar extent. Cell growth inhibition of these inhibitors is well established. Taken together, these results strongly suggest that CyP facilitate HPV16 and HPV18 infection.

### siRNA mediated knock down of CyP

**Figure 2:**
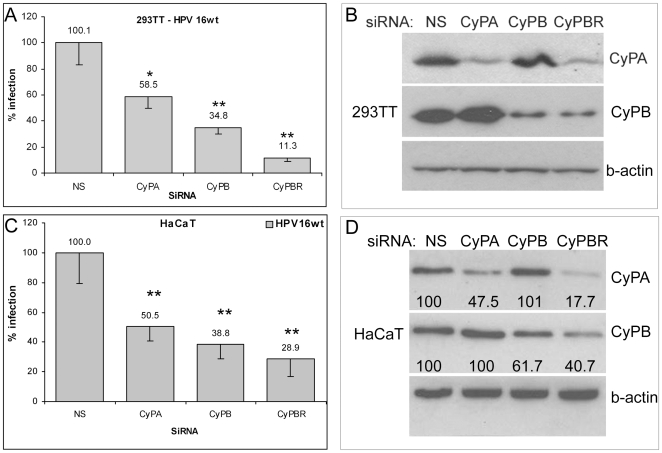
Knock down of CyP inhibits HPV16 infection. 293TT (A,B) or HaCaT (C,D) cells were transfected with indicated siRNA and infected with HPV16 pseudovirus 48h after transfection. Infection was scored at 72 hpi. Representative graphs based on six replicates are shown (A,C). Knockdown of CyPA and CyPB was confirmed by Western blot prior to infection (B,D). Numbers indicate percent levels of protein after correcting for input (D). *: p<0.05; **: p<0.01.

In order to determine, which CyP family member may be involved and to confirm our findings, we used an siRNA approach to knock down individual CyP. First, we used an siRNA, si-CyP[broad], which has been shown to target several members of the CyP family including CyPA, CyPB, CyPE, and CyPH [11]. 293TT cells were transfected with si-CyP[broad] 48 prior to infection with HPV16. Western blot confirmed the significant reduction of steady state CyPA and CyPB protein levels (Figure 2B) and infection was reduced to 11% (p<0.01) compared to cells transfected with a control siRNA (Figure 2A). Individual knock down of CyPA and CyPB with specific validated siRNAs [11] also reduced infection to 59% (p<0.05) and 35% (p<0.01), respectively. Specificity of the siRNA knock down for their target was confirmed by Western blot (Figure 2B). The data indicated that both CyP may play a role in HPV16 infection. Compared to CyPA, knockdown of CyPB consistently resulted in stronger inhibition (p<0.05). Similar results were obtained for HaCaT cells. However, due to reduced transfection efficiency of HaCaT cells (70% vs. 95% for 293TT) the inhibitory effect was not as pronounced (Figure 2C and 2D).

### Internalization of viral capsids in presence of CyP inhibitors

**Figure 3:**
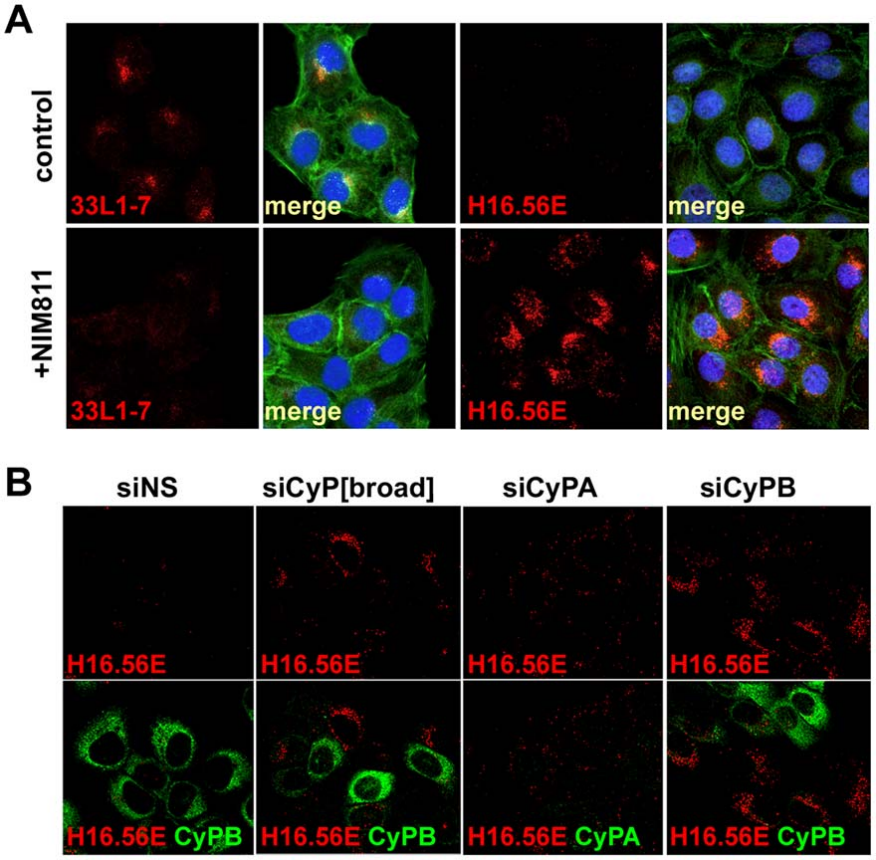
Inhibition of CyP leads to noninfectious HPV16 internalization. (A) HaCaT cells were infected with HPV16 pseudovirus in the absence or presence of NIM811. At 18 hpi cells were fixed and stained using conformation-dependent NmAb H16.56E or linear epitope-specific nonneutralizing 33L1-7. Cells were also stained for actin and DNA using AF^488^–labeled phalloidin (green) and Dapi (blue), respectively. Images were taken with a Leica DBMI6000 at 40× magnification. (B) HaCaT cells were transfected with indicated siRNA. At 48 hpTx, cells were harvested and reseeded for infection with HPV16 pseudovirions. At 18 hpi cells were fixed and stained using H16.56E (red) and CyPA or CyPB (green). Images were taken with a confocal Zeiss LSM 510 microscope at 63× magnification.

To identify the stage at which infection is blocked by CyP inhibitors, we first measured internalization using immunofluorescence (IF). It was shown by several groups that most surface-exposed conformational epitopes that are recognized by neutralizing monoclonal antibodies (NmAb) are destroyed following entry and that L1 protein segregates from the L2/DNA complex in acidic endocytic compartments [18],[29],[30]. During this process reactivity of antibodies specific for hidden linear L1 epitopes is gained [32]. We used NmAb H16.56E to determine if conformational epitopes are lost in the presence of NIM811. H16.56E binding site includes but is not restricted to the N-terminal portion of the FG loop (HPV16 L1 residues 260–270) [33]. We also used mAb 33L1-7, which binds a linear epitope (residues 303–313) that is neither accessible in capsomeres nor in intact particles [34],[35] and recognizes L1 protein late in HPV entry [32]. In untreated cells at 18 h post infection (hpi) with HPV16 pseudovirus, H16.56E reactivity was hardly detectable but perinuclear 33L1-7 staining was obvious indicative of particle internalization and accessibility of the 33L1-7 epitope (Figure 3A). In contrast, we observed a strongly increased perinuclear signal with H16.56E when infection was performed in the presence of 10 µM NIM811. The signal for 33L1-7 was greatly diminished under these conditions (Figure 3A). Similar results were obtained when NIM811 was replaced by CsA (data not shown). We will use the term ‘stabilized capsid phenotype’ to describe the increased reactivity of internalized pseudovirions with H16.56E. These data demonstrate that, first, viral particles are indeed internalized in the presence of CyP inhibitor and, second, the conformational L1 epitope recognized by H16.56E is stabilized.

We again used siRNA knock down to identify the CyP family member responsible for the stabilized capsid phenotype. For this, HaCaT cells were transfected with unspecific control siRNA, si-CyP[broad], si-CyPA or si-CyPB 48 h prior to infection with HPV16 pseudovirus. Successful down regulation of CyPB was confirmed by IF (Figure 3B). Down regulation of CyPA could not be determined by IF because of lack of CyPA-specific antibody reactivity in this assay. However, successful transfection was monitored using FITC-labeled siRNA (not shown) and knock down of CyPA was confirmed by Western blot. Cells with reduced levels of CyPB following transfection with si-CyPB or si-CyP[broad] displayed a stabilized capsid phenotype at 18 hpi, whereas adjacent cells, which were not transfected as indicated by strong staining for CyPB, showed much less reactivity with H16.56E (Figure 3B). A stabilized capsid phenotype was not detected in cells transfected with si-CyPA, even though basal level of reactivity with H16.56E is evident. Taken together these data suggest that blockage of CyPB activity may be responsible for the stabilized capsid phenotype.

### Effect of CyP inhibitors on L2 conformational changes

**Figure 4:**
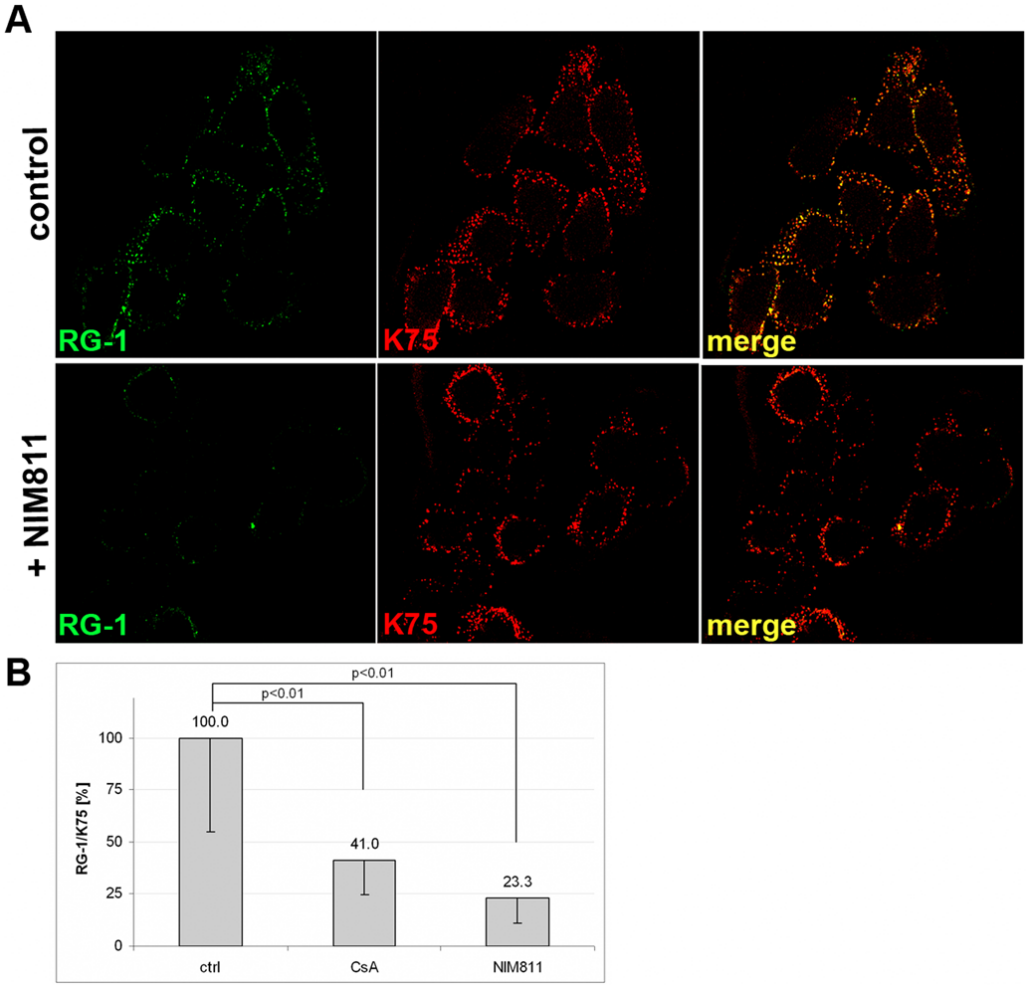
CyP facilitate exposure of the RG-1 epitope after cell attachment. HPV16 pseudovirus was bound to HaCaT cells for 2 h at 4°C and chased for 4 h at 37°C in presence or absence of NIM811. (A) Cells were subsequently stained with L2-specific RG-1 and L1-specific K75 antibody. All images were taken using the same settings. (B) RG-1– and K75–specific signal strength of randomly selected cells (n>15 for each group) was measured. RG-1 reactivity normalized to K75 signal strength is plotted after subtraction of background signal. The graph shows quantifications from one experiment. However, the experiment was repeated three times with similar outcomes.

Previously we observed a stabilized capsid phenotype when transfer to secondary receptors on the cell surface was blocked by antibodies or drugs [18]. Furthermore, CyPB is found on the cell surface where it is associated with HSPG [6],[36]. Therefore, we hypothesized that CyPB may facilitate the conformational shifts reported for both capsid proteins upon interaction with cell surface HSPG [23],[25]. Currently, the only reliable test for these changes measures the exposure of the L2 N-terminus using the L2-specific NmAb RG-1. RG-1 binds to a peptide encompassing HPV16 L2 residues 17 to 36 [37]. RG-1 reactivity with L2 protein incorporated into virions requires cell attachment-induced exposure of the L2 N-terminus and furin cleavage [24]. To test the role of CyP in conformational shifts, HPV16 pseudovirus was bound to HaCaT cells for 2 h at 4°C and was chased for 4 h at 37°C prior to cell surface staining with RG-1 (a kind gift of R.B. Roden, John Hopkins University) and K75 polyclonal VLP antisera. In control infection we found strong RG-1 signal, which perfectly overlapped with cell-associated L1-specific K75 binding (Figure 4A). RG-1 reactivity was greatly diminished albeit not completely abolished when HaCaT cells were infected in the presence of NIM811 (Figure 4A), whereas reactivity with K75 was not decreased. Similarly, CsA treatment decreased RG-1 signal albeit not as pronounced (data not shown). We quantified the RG-1- and K75-specific signals using software provided by Zeiss and found statistically significant reductions of over 70% and 59% of relative RG-1 signal strength in presence of NIM811 and CsA, respectively (p<0.01) (Figure 4B). Taken together, these data strongly suggest that CyPB activity is required for exposure of the RG-1 epitope on the viral capsid and lend support for a function of cell surface CyPB in HPV16 infection.

**Figure 5:**
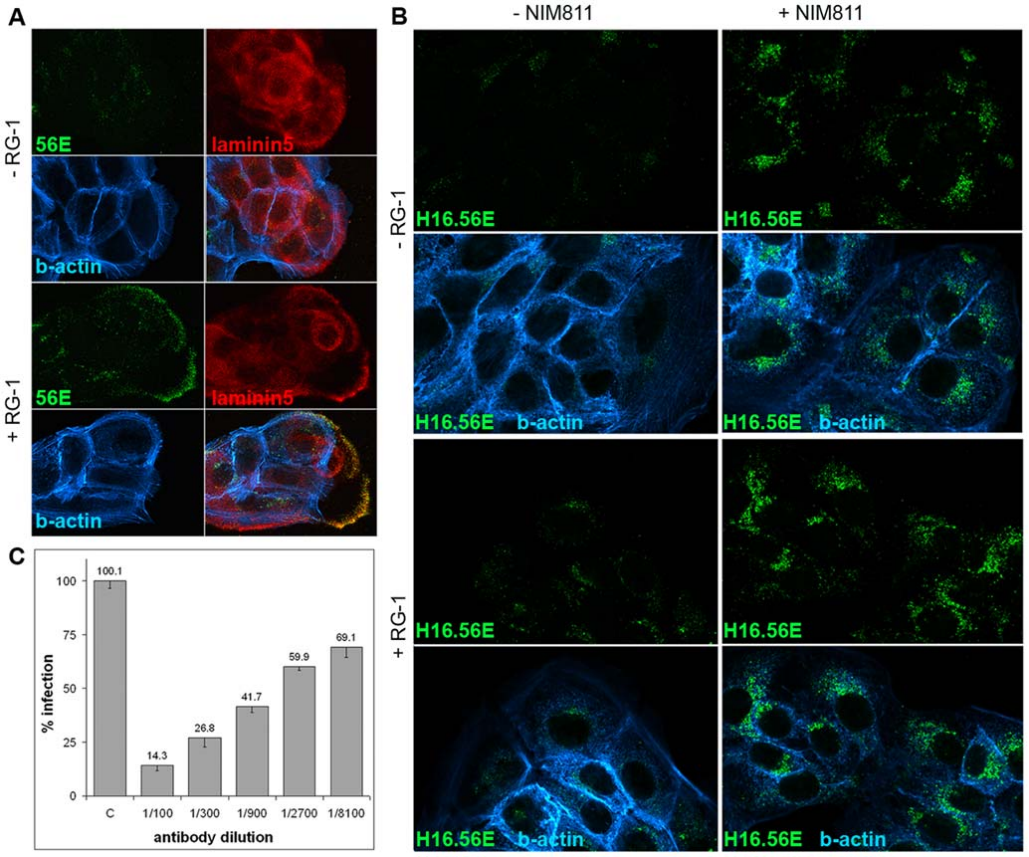
Effect of RG-1 on particle internalization. (A) HPV16 pseudovirus was added to HaCaT grown on coverslips and incubated for 18 h at 37°C in the presence or absence of RG-1 (1∶10 dilution of cell culture supernatant). Samples were stained for viral particles and ECM using H16.56E and rabbit polyclonal antiserum specific for laminin 5, respectively. Note the accumulation of viral particles on ECM in presence of RG-1. As fixation prior to addition of secondary antibody destroys reactivity with RG-1 [24, and our own observations], the signal picked up by mouse-specific secondary antibody is solely attributable to H16.56E. (B) HaCaT cells were grown and infected as above with the addition of NIM811 during the incubation where indicated. Samples were stained with H16.56E. Note that the presence of RG-1 did not affect the stabilized capsid phenotype and viral internalization irrespective of NIM811 treatment. Images were taken with a Zeiss LSM at 63× magnification focusing on ECM (A) and cells (B), respectively. Actin staining with labeled phalloidin is shown in blue. (C) Neutralization of HPV16 by RG-1 at indicated dilutions was measured at 72 hpi of 293TT cells (n = 5).

Recently, it has been shown that the presence of RG-1 antibody during infection of HaCaT cells with HPV16 pseudovirions prevents infection and virus internalization and relocates viral particles from the cell surface to ECM [24]. We took advantage of this observation to strengthen our findings. We reasoned that, irrespective of presence of RG-1, viral particles should still be internalized and display a stabilized capsid phenotype in presence of NIM811, if the RG-1 epitope is indeed not accessible to antibody binding after drug treatment. To test this, HPV16 pseudovirus was bound to HaCaT cells for 2 h at 4°C in the presence or absence of this drug. After washout of unbound virus, cells were incubated overnight in presence of NIM811 and RG-1. Confirming previous findings [24], RG-1 treatment alone induced deposition of the majority of viral particles to ECM in the absence of NIM811, as evidenced by colocalization of capsid-specific H16.56E signal with the ECM marker Laminin 5 (Figure 5A). We also confirmed the neutralizing capacity of RG-1 using 293TT cells (Figure 5C) to ascertain that this antibody is functional in our hands. Inhibition of HPV16 pseudovirus infection by this antibody using HaCaT cells was previously demonstrated by others [24],[37]. However, the presence of RG-1 antibody in addition to drugs did not prevent internalization of viral capsids, as evidenced by a stabilized capsid phenotype (Figure 5B) and did not result in increased deposition of viral particles on ECM (not shown). It should be noted that RG-1 treatment in the absence of NIM811 displayed a weak but reproducible stabilized capsid phenotype (Figure 5B) suggesting that not all particles are displaced from the cell surface and are instead internalized in a noninfectious manner. These data further support our notion that CyPB action on the cell surface is required for the conformational change resulting in exposure of the RG-1 epitope, which is a prerequisite for infectious internalization.

### A putative binding site for CyP at the L2 N-terminus

**Figure 6:**
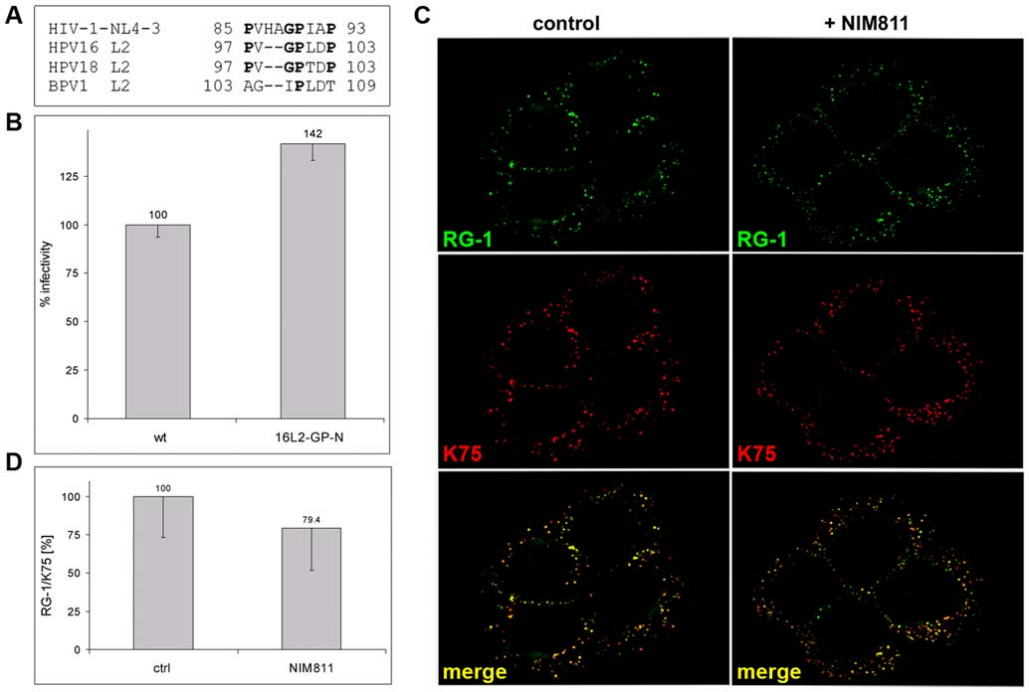
L2 protein is the likely target of CyPB. (A) Sequence alignment of selected PV L2 proteins with CyPA binding site of HIV capsid protein. (B) 293TT cells were infected with similar amounts of HPV16 wt and 16L2-GP-N mutant pseudovirus and scored at 72 hpi. The difference in infectivity is statistically significant (p<0.01; n = 5) based on testing two independent pseudovirus preparations. (C) 16L2-GP-N mutant pseudovirus was bound to HaCaT cells in presence or absence of NIM811 for 4 h at 37°C and subsequently stained with RG-1 and K75. All images were taken with the same settings. (D) Quantification of RG-1 and K75 signal strength using randomly selected cells (n>15).

**Figure 7:**
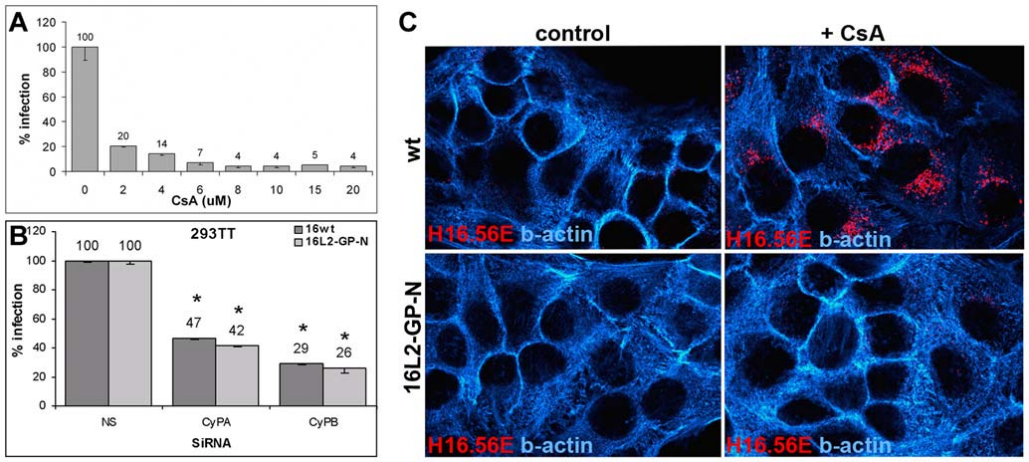
Mutant pseudovirus infection is impaired by CyP inhibitors. (A) Sensitivity of 16L2-GP-N mutant pseudovirus to CsA was determined by infection of 293TT cells. (B) 293TT cells were transfected with indicated siRNA and infected with mutant pseudovirus at 48 hpTx and scored at 72 hpi. *: p<0.01. (C) HaCaT cells grown on coverslips were infected with HPV16 wt or 16L2-GP-N mutant pseudovirus. At 18 hpi cells were stained with H16.56E (red) and labeled phalloidin (blue).

Not much information is available regarding CyPB substrate binding sites. However, CyPA binding to the HIV capsid protein has been mapped to 85-PXXXGPXXP-93, which is located between Helix 4 and 5 [7]. We found similar sequence elements at the N-terminus of L2 conserved among many but not all members of the *Papillomaviridae* family (Figure 6A). We exchanged glycine and proline residues of L2 at positions 99 and 100 within the putative CyP binding site for alanine to test their importance for HPV16 infection. We hypothesized that this mutant is either defective for infection due to loss of CyP binding or does not require active CyP for exposure of the L2 N-terminus due to higher flexibility in this L2 region induced by amino acid exchanges. We found that 16L2-G99A-P100A (16L2-GP-N) is incorporated into particles similar to wt L2 (not shown). Mutant pseudovirus retains full infectivity in 293TT (Figure 6B) and HaCaT cells (data not shown), which is consistently and statistically significantly increased compared to wt (p<0.01). When we bound 16L2-GP-N mutant pseudovirus to HaCaT cells and surface-stained with RG-1 and K75 after a 4 h chase at 37°C, we observed similar reactivity of RG-1 with cell-bound pseudovirions in absence or presence of NIM811 (Figure 6C). Quantitative analysis of signal strength confirmed that reactivity of RG-1 with mutant pseudovirus is not significantly reduced by this drug (Figure 6D) in contrast to wt pseudovirus (Figure 4). These data suggested that 16L2-GP-N mutant pseudovirus does not require CyP activity for exposure of the RG-1 epitope. Nevertheless, infection was still sensitive to CsA (Figure 7A) and siRNA knock down of CyP (Figure 7B). However, unlike wt pseudovirus mutant pseudovirus did not produce the stabilized capsid phenotype after treatment with drugs (Figure 7C) or siRNA knock down of CyP (not shown), although H16.56E was still able to detect mutant viral particles on the cell surface and on ECM (data not shown). Taken together, these data indicate not only that 16L2-GP-N mutant pseudovirus bypasses the requirement for cell surface CyPB but also that HPV16 infection requires CyP at a second, possibly intracellular, stage of entry and transport. Furthermore, they strongly support our previous notion that, in presence of CyP inhibitors, wt virus is shunted into a noninfectious entry pathway.

To determine whether the requirement for CyP is a conserved feature among papillomaviruses we tested a number of low and highrisk HPV types as well as BPV-1 for sensitivity to CsA. We found that HPV6, HPV45 and HPV58 were inhibited by CsA similar to HPV16 and HPV18, whereas BPV1, HPV5, HPV31, and HPV52 were relatively resistant to CsA (Table 1). These data suggest that different papillomavirus types have different requirements for CyP, which may be reflective of the entry strategies these viruses evolved.

**Table 1:** Sensitivity of PV types to CsA

*PV type*	*2 µM CsA*	*10 µM CsA*
**6**	45.3 (7.4)	11.9 (2.1)
**45**	70.4 (1.9)	20.9 (1.1)
**58**	63.8 (10.4)	20.7 (6.8)
**5**	93.7 (0.9)	83.3 (2.7)
**31**	93.4 (2.7)	51.7 (5.6)
**52**	80.4 (9.0)	73.5 (7.0)
**BPV1**	81.0 (7.8)	68.4 (17.6)

Values represent percent infection with standard deviations in parentheses. Values are based on five replicates with the exception of HPV5 and HPV52 (three replicates).

## Discussion

Here, we report that CyP facilitate infection of the oncogenic HPV16 and 18 among other HPV types. Focusing on HPV16 and using specific drugs, siRNA knock down and mutant pseudovirus we provide evidence that CyP are required at two different stages following primary attachment to host cells. In addition, siRNA knock down data point to the involvement of two members of the CyP family in the infection process: CyPA and CyPB. Combined knock down using siCyP[broad] affected infection more severely than individual knock downs suggesting they may facilitate different steps of HPV16 infection. Our data indicate that CyPB is functioning on the cell surface. However, we were not yet able to identify the step requiring CyPA. Also, at the moment we cannot completely rule out the involvement of additional CyP family members, like CyPE and CyPH, whose expression should also be affected by siCyP[broad].

We provide evidence that cell surface CyPB is essential for triggering events that lead to infectious internalization of viral particles probably by catalyzing conformational changes of viral capsid proteins. It is well established that both HPV16 capsid proteins undergo conformational changes on the cell surface prior to internalization. Conformational changes induced in L1 are not well defined but seem to involve the BC loop [23]. Conformational changes induced in L2 protein result in exposure of some forty N-terminal amino acids, which allows furin convertase-mediated cleavage of L2 and binding of the L2-specific NmAb RG-1 [24],[25],[38]. CyP inhibition greatly reduced exposure of the RG-1 epitope following cell attachment as measured directly by IF and indirectly by determining the fate of cell bound pseudovirus in the presence of CyP inhibitors and RG-1. This strongly indicates that CyP activity is required to make the RG-1 epitope accessible to antibody binding. However, the block was not complete and residual reactivity with RG-1 was observed in presence of inhibitors, which could possibly be attributed to the presence of activated particles in the pseudovirus preparation [18],[28] and/or to the baseline spontaneous conformational change in absence of CyP activity due to receptor engagement. Nevertheless, the reduction in RG-1 reactivity by CyP-specific drugs was found to be correlated with reduction of infectivity by drugs.

We also provide evidence that L2 protein may be the substrate for CyP. First, we were able to bypass the requirement for cell surface CyP by introducing amino acid changes in a putative CyP binding site within L2, which is accessible in mature virions [39]. Mutant pseudovirus did not require CyP activity for exposure of L2 as demonstrated by IF. However, at this point we cannot completely rule out that CyP functions rather indirectly by (i) modifying cell surface receptors, since CyP have been shown to isomerize prolyl peptide bonds of cell surface markers, thus modifying their biological function [40],[41], and by (ii) regulating cell trafficking and cell surface expression of proteins [42]. However, this is rather unlikely since BPV1, which uses the same route of internalization as HPV16 [43],[44], is not blocked by CyP inhibitors. Second, specific blockage of CyPB induced noninfectious internalization with the hallmark of a stabilized capsid phenotype. In this respect CyPB inhibition is similar to post-attachment treatment with the BC loop-specific antibody H33.J3, heparinase, or the HS binding drug DSTP-27, which also induce noninfectious internalization and stabilization of viral capsids [18]. It was suggested that these treatments all block secondary receptor interactions, which seems to require an exposed L2 N-terminus [18]. It is unlikely that L1 rather than L2 protein is the substrate of CyP. This is based on our unpublished observations that CsA, NIM811 or CyP-specific siRNAs do not block L1 conformational changes occurring on the cell surface.

Interestingly, mutant 16L2-GP-N pseudovirus remained sensitive to CyP inhibitory drugs and siRNA knock down. However, bypassing the need for cell surface CyPB using mutant pseudovirus yielded an inhibition phenotype distinct from wt particles. We no longer observed capsid stabilization. This suggests that CyP activity is required at a subsequent step during internalization and/or intracellular transport. So far, we were not able to identify the exact step(s) that require CyP activity and therefore cannot predict which specific CyP family member may be involved. However, a second putative CyP binding site is located near the C-terminus of L2 (409-**P**LVS**GP**DI**P**-417). This sequence is close to a region that has been shown to mediate interaction of L2 with L1 capsomeres in HPV11 [45]. It is therefore tempting to speculate that endocytic CyP mediates segregation of L2 from L1. As the C-terminal section of L2 is required for membrane destabilization and passage of membranes [30] as well as for interaction with dynein [46], this could free the C-terminus allowing association and penetration of surrounding membranes and consequently the L2/DNA complex to egress from endosomes and retrograde transport towards the nucleus. CyPB encodes a signal peptide and is therefore found in the luminal compartment of intracellular membranes making it a likely candidate. However, CyPA is also secreted into the extracellular space, even though it lacks a signal peptide, suggesting it finds its way into the luminal compartment of at least the secretory pathway.

Host cell CyP do not facilitate infection of all papillomaviruses. Support for this notion came from our finding that HPV5, HPV31, HPV52, and BPV1 are rather resistant to CyP-specific drugs. This may reflect the evolution of different internalization strategies. For example, HPV31 is internalized via caveolae-dependent endocytosis [31],[47] whereas HPV16 uses a caveolae- and clathrin-independent pathway [32]. BPV1 L2 does not harbor putative CyP binding sites and replacing key proline residues by more flexible amino acids may make a catalytic activity dispensable for L2 exposure, as we have shown with HPV16L2-GP-N. The entry pathways of HPV5 and HPV52 have not been investigated yet. However, the L2 protein of both HPV types has a putative N-terminal CyP binding site.

Attachment-induced conformational changes are a common theme in virus infection. They are usually triggered by interaction with specific receptors, which allows interaction with secondary receptors or, more often, trigger cell fusion events. Although chaperones present in endocytic vesicles or the endoplasmic reticulum have been shown to facilitate virus uncoating and translocation across membranes [48],[49], this is the first report to implicate chaperones in mediating conformational changes of capsid proteins on the surface of target cells.

With this report we are adding another virus family to the list of viruses dependent on CyP activity for completion of their life cycle. Despite 15 years of study, the role of CyPA in HIV-1 infection is not yet fully defined (for review see [7],[50]). Similarly, its involvement in MCMV infection of neural progenitor cells has not been characterized in molecular detail [12], whereas it was convincingly demonstrated for HCV that ER-resident CyPB enhances the RNA binding activity of the NS5B RNA polymerase and consequently genome amplification [11]. With the identification of CyPB as modifier of oncogenic HPV capsid protein conformation, which activates the virus for entry via an infectious pathway, for the first time we have characterized its role at the molecular level during cell surface events of viral infections. This should allow characterizing the complex events preceding internalization in more detail and adds a putative drug target for prevention of HPV-induced diseases, especially since CsA has been approved for and is already being used in clinical settings.

## Materials and Methods

### Cell lines, plasmids, antibodies, and pseudovirions

293TT cells and expression plasmids for codon-optimized structural genes coding for HPV5, HPV6, HPV18, HPV31, HPV45, HPV52, HPV58 as well as BPV1 were kindly provided by John Schiller and Chris Buck, Bethesda [27],[51]. Codon-optimized HPV16 L1 and L2 expression plasmids were a kind gift from Martin Müller, Heidelberg [52]. HPV16L1-specific rabbit polyclonal antisera K75, mouse monoclonal antibody H16.56E and 33L1-7 have been described previously [34],[35]. Anti-CyPA polyconal rabbit antibody was obtained from Dharmacon (cat #: 07-313). CyPB polyclonal rabbit antibody was purchased from Affinity BioReagents Inc (Golden, Colorado; cat #: PA1-027). However, we noticed that only lot number 328-120 and prior lots were reactive in IF. All subsequent lots tested were not reactive in IF analyses. Laminin 5 rabbit polyclonal antibody was from Abcam (cat #: ab14509). AF^488^-labeled GFP-specific rabbit polyclonal antibody was obtained from Invitrogen. Mouse monoclonal L2-specific RG-1 antibody was kindly provided by Richard Roden, John Hopkins University, Baltimore. AlexaFluor (AF)–labeled secondary antibodies and phalloidin were purchased from Invitrogen. Pseudovirions were generated and purified using Optiprep gradient centrifugation following published procedures [27]. Pseudovirus yield was determined by green fluorescent protein (GFP)–specific quantitative real time polymerase chain reaction (qRT–PCR).

### Inhibitors and reagents

Cyclosporin A was obtained from Toronto Research Chemicals (cat #: C988900). NIM811 was a kind gift from Novartis. Verapamil (cat #: 676777), Nifedipine (cat #: 481981), 11R-VIVIT (cat #: 480401) and INCA-6 (cat #: 480403) were obtained from Calbiochem. FK 506 (cat #: F1030) was purchased from A.G. Scientific (San Diego). The cell viability and proliferation assay ‘CellTiter96 Aqueous One Solution’ was purchased from Promega (Madison, WI). This assay measures the quantity of formazan product, which is directly proportional to the number of living cells.

### Infection assay

293TT cells were seeded a day before and allowed to attach. Next day, drugs were serially diluted in complete DMEM in 24 well-plates and adequate amounts of pseudoviruses were added to achieve infection levels of 10 to 30%. Infectivity was scored by counting GFP expressing cells at 72 hpi using flow cytometry. Similar protocol was followed for infection assay using HaCaT and HeLa cells except that cells were fixed with 2% paraformaldehyde, permeabilized with 0.2% Triton X-100 in phosphate buffered saline (PBS), stained with AF^488^-labeled GFP-specific antibody and counted using a Leica DMBI 6000 fluorescence microscope. Unless otherwise stated standard deviation was based on at least five replicates from at least two independent experiments.

### RNA interference

RNA interference was carried out using synthetic siRNA duplexes with symmetric 3′-deoxythymidine overhangs. siRNA duplexes si-CyPA, 5′-AAGCATA CGGGTCCTGGCATC-3′; si-CyPB, 5′-AAGGTGGAGAGCACCAAGACA-3′; and si-CyP(broad), 5′-AAGCATGTGGTGTTTGGCAAA-3′), which have been described and validated before [11], were purchased from Integrated DNA Technologies Inc. Non-specific siRNA, si-NS, 5′-AAGTCCGTGCCGTCAGTTCTCAGAA-3′ was obtained from Invitrogen. Cells were transfected with 3 µg of siRNA duplexes in serum-free medium using MATra reagent (IBA biotagnology, Goettingen; cat. #: 7-2001-100) according to manufacturer's protocol. Typical siRNA transfection efficiency was found to be 70% for HaCaT and 95% for 293TT cells as monitored by fluorescein-labeled control siRNA duplex. CyP knockdown was confirmed 48 h post siRNA transfection (hpTx) by Western blot.

### Infection and immunofluroscence assay after siRNA knockdown of CyP

HaCaT and 293TT cells were transfected with siRNA as mentioned above. 48 hpTx, HaCaT were harvested with trypsin and reseeded onto cover slip for immunofluorescence study. Few hours later, when cells had attached, they were infected. At 18 hpi samples were fixed with 4% paraformaldehyde and stained. Alternatively, cells were incubated for 72 h and subsequently stained for GFP as described above to score infection [18]. For infection assay using 293TT, cells were harvested, reseeded into 96 well plates and allowed to attach. Few hours later, they were infected and scored at 72 hpi by counting GFP positive cells.

### Immunofluorescence in presence of CyP inhibitor

HaCaT cells were grown on cover slips till ∼50% confluency and infected with HPV16 pseudovirus in presence of NIM811, antibody, or DMSO. At the indicated times post infection, cells were washed with PBS and fixed with 4% paraformaldehyde for 15 min at room temperature, washed, permeabilized with 0.2% Triton X-100 in PBS for 2 min, washed, and blocked with 5% goat serum in PBS for 30 min, followed by a 1 h incubation with primary antibodies at 37°C. After extensive washing, cells were incubated with AlexaFluor-tagged secondary antibodies and fluorescently labeled phalloidin for 1 h. After extensive washing with PBS, cells were mounted in ‘Gold Antifade’ containing Dapi (Invitrogen). Images were captured by confocal microscopy (Zeiss 510 Laser Scanning Confocal Microscope operated by LaserSharp2000 software) or by standard fluorescence microscopy (Leica DMBI 6000 microscope). Within individual experiments the same microscope settings and exposure times were used. For quantification of fluorescent signal intensity, the LSM server software provided with the confocal microscope was used. Signal strength was acquired from randomly selected single cells (n>15 for each group). The average region of interest was not significantly different among all groups. Background was determined using mock infected cells and subtracted prior to calculations.

RG-1 staining was performed as described [24]. In brief, infected HaCaT cells were shifted to 4°C and incubated with RG-1 and K75 for 1 h in presence of 2% normal goat serum. After extensive washing and incubation with fluorescently labeled secondary antisera, cells were fixed for 20 min in 2% paraformaldehyde. After washing, cells were incubated for 5 min with phalloidin-AF^647^ conjugate and mounted.

### Accession numbers for genes and proteins mentioned in the text

CyPA: NM_021130; CyPB: NM_000942; codon optimized HPV16 L1: AJ313179; codon optimized HPV16 L2: AJ313180
